# *Litsea japonica* Leaf Extract Suppresses Proinflammatory Cytokine Production in Periodontal Ligament Fibroblasts Stimulated with Oral Pathogenic Bacteria or Interleukin-1β

**DOI:** 10.3390/ijms19092494

**Published:** 2018-08-23

**Authors:** In-Gyeong Yun, Sun-Hee Ahn, Weon-Jong Yoon, Chang Sook Kim, Yun Kyong Lim, Joong-Ki Kook, Seunggon Jung, Choong-Ho Choi, Tae-Hoon Lee

**Affiliations:** 1 Department of Preventive and Public Health Dentistry, Dental Science Research Institute, School of Dentistry, Chonnam National University, Gwangju 61186, Korea; heavenstairs@naver.com (I.-G.Y.); hochoi@chonnam.ac.kr (C.-H.C.); 2 Department of Oral Biochemistry, Dental Science Research Institute, School of Dentistry, Chonnam National University, Gwangju 61186, Korea; sun3193@jnu.ac.kr; 3 Jeju Biodiversity Research Institute (JBRI), Jeju Technopark (JTP), Jeju 63608, Korea; yyjkl@jejutp.or.kr (W.-J.Y); cskim@jejutp.or.kr (C.S.K.); 4 Department of Oral Biochemistry, School of Dentistry, Chosun University, Gwangju 61452, Korea; dbsruddl77@hanmail.net (Y.K.L); jkkook@chosun.ac.kr (J.-K.K.); 5 Department of Oral and Maxillofacial Surgery, School of Dentistry, Chonnam National University, Gwangju 61186, Korea; ashgray79@gmail.com; 6 Department of Molecular Medicine (BK21plus), Chonnam National University Graduate School, Gwangju 61186, Korea

**Keywords:** periodontal disease, inflammation, periodontal ligament, *Litsea japonica* leaf extract, IL-6, IL-8

## Abstract

Periodontal disease, a chronic disease caused by bacterial infection, eventually progresses to severe inflammation and bone loss. Regulating excessive inflammation of inflamed periodontal tissues is critical in treating periodontal diseases. The periodontal ligament (PDL) is primarily a connective tissue attachment between the root and alveolar bone. PDL fibroblasts (PDLFs) produce pro-inflammatory cytokines in response to bacterial infection, which could further adversely affect the tissue and cause bone loss. In this study, we determined the ability of *Litsea japonica* leaf extract (LJLE) to inhibit pro-inflammatory cytokine production in PDLFs in response to various stimulants. First, we found that LJLE treatment reduced lipopolysaccharide (LPS)-induced pro-inflammatory cytokine (interleukin-6 and interleukin-8) mRNA and protein expression in PDLFs without cytotoxicity. Next, we observed the anti-inflammatory effect of LJLE in PDLFs after infection with various oral bacteria, including *Fusobacterium nucleatum*, *Porphyromonas gingivalis*, *Treponema denticola*, and *Tannerella forsythia*. These anti-inflammatory effects of LJLE were dose-dependent, and the extract was effective following both pretreatment and posttreatment. Moreover, we found that LJLE suppressed the effect of interleukin-1 beta-induced pro-inflammatory cytokine production in PDLFs. Taken together, these results indicate that LJLE has anti-inflammatory activity that could be exploited to prevent and treat human periodontitis by controlling inflammation.

## 1. Introduction

Periodontitis is one of the most common oral infectious diseases; it is an inflammatory disease caused by oral pathogens, which destroy the periodontal ligament (PDL) and alveolar bone with gingival recession [1,2,3]. The PDL is a fibrous connective tissue between the cementum and alveolar bone surrounding the root; it plays an important role in the maintenance, function, and regeneration of the periodontal tissue, as well as in the regulation of periodontal inflammation [4]. Periodontal tissues are known to be sensitive to lipopolysaccharide (LPS) and other pathogenic factors [5].

Conversion from healthy periodontal status to disease is not caused by a single bacterium, but by bacterial complexes of certain species. For example, Socransky and Haffajee stratified microbial populations into five groups or complexes that appear to co-occur and represent a bacterial consortium associated with the development of biofilm-associated infections, gingivitis, and periodontitis [6,7,8]. In particular, a bacterial complex named “red complex” that consists of *Porphyromonas gingivalis*, *Treponema denticola*, and *Tannerella forsythia* has been shown to increase in deep periodontal pockets, and is strongly related to the progression of periodontal disease in advanced periodontal lesions [9,10,11]. In addition, *Fusobacterium nucleatum* plays a role in the development of periodontitis by acting as a bridge for the interaction of red complex bacteria [12,13]. Recently, we reported the importance of *F. nucleatum* in the periodontal microbial ecology and host response to *F. nucleatum* [14,15].

Oral bacteria and their components, including LPS, promote the production of pro-inflammatory cytokines in PDL fibroblasts [4,16]. Interleukin-6 (IL-6) and interleukin-8 (IL-8) are prominent pro-inflammatory cytokines that are closely related to periodontitis, leading to periodontal tissue destruction and alveolar bone resorption [17,18]. These pro-inflammatory cytokines are mainly produced by immune cells, i.e., macrophages and lymphocytes, when they are exposed to inflammatory stimuli [19], and by gingival fibroblasts and the PDL, which functions as a physical barrier after stimulation with bacterial components or inflammatory mediators, including IL-1β [20,21,22]. Thus, many studies have been focusing on identifying molecules, synthetic chemicals, or natural compounds that can suppress the production of inflammatory cytokines to control periodontal inflammation [23,24,25].

Recently, concerns have been raised about the use of synthetic drugs owing to their adverse effects and toxicity [26]. For example, 8% of hospital admissions in the US were reported to be caused by the adverse effects of synthetic drugs [27]. Thus, natural products, which are relatively safe and inexpensive, have been proposed as alternatives, and are considered to be free from undesirable adverse effects [28,29,30]. Moreover, deaths or hospitalizations in the US due to herbs are extremely rare [26]. Although the potential adverse effects and cellular toxicity of plant-derived natural products need to be elucidated, they are generally considered to be safe and effective. Korean traditional medicine has long relied on the use of natural products for the therapy or prevention of diseases [31,32].

*Litsea japonica* is an evergreen broadleaf species belonging to the family, Lauraceae. It is found in Jeollanamdo, Gyeongsangnamdo Island, Jeju, and Ulleungdo in Korea and in South Asian regions, including Japan. Numerous studies have shown that the extracts of *L. japonica* fruits have anti-inflammatory [33] and anti-diabetic effects [34], can protect against diabetic retinopathy [35], and can prevent osteoarthritis [36]. Although several biological activities of *L. japonica* extract have been reported, the effect of its leaf extract on periodontal disease has not yet been investigated. This study aimed to investigate the effect of *L. japonica* leaf extract (LJLE) on inflammation induced by various inflammatory stimuli, including LPS, several strains of oral bacteria, and IL-1β, in the PDL.

## 2. Results

### 2.1. Effect of LJLE on LPS-Induced Pro-Inflammatory Cytokine Expression in PDLFs

**Figure 1:**
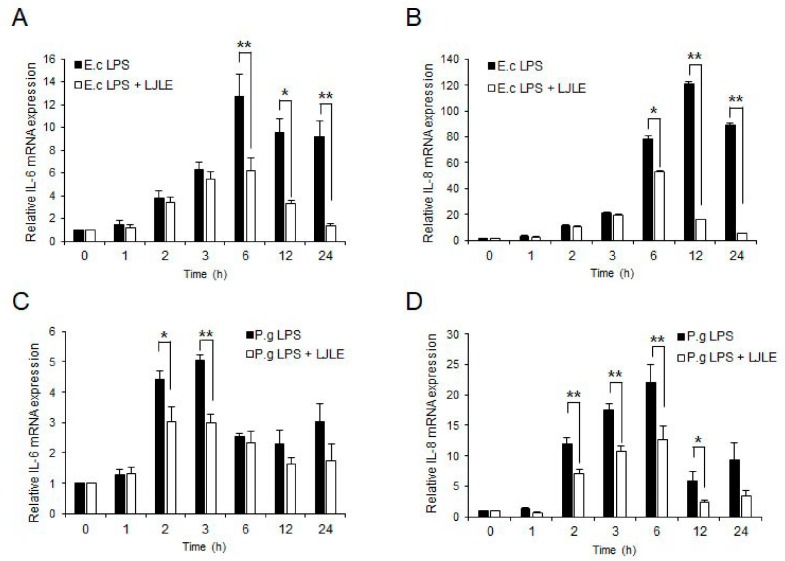
*Litsea japonica* leaf extract (LJLE) inhibits pro-inflammatory cytokine expression in lipopolysaccharide (LPS)-stimulated human periodontal ligament fibroblasts (PDLFs). Human PDLFs were stimulated with LPS in the absence or presence of 100 μg/mL LJLE for different times (0, 1, 2, 3, 6, 12, and 24 h). Real-time polymerase chain reaction (PCR) was performed as described in the Materials and Methods. (**A**,**B**) Time-course effects of LJLE on IL-6 and IL-8 mRNA in 1 ng/mL *Escherichia coli* (E.c) LPS-stimulated PDLFs. (**C**,**D**) Time-course effects of LJLE on IL-6 and IL-8 mRNA in 10 ng/mL *Porphyromonas gingivalis* (P.g) LPS-stimulated PDLFs. * *p* < 0.05 and ** *p* < 0.01 compared with LPS alone (unpaired two-tailed Student’s *t*-test).

To investigate whether LJLE affects LPS-induced pro-inflammatory cytokine production in periodontal ligament fibroblasts (PDLFs), we determined its effects on IL-6 and IL-8 mRNA expression in LPS-stimulated PDLFs. First, we determined the concentration- and time-dependent response of pro-inflammatory cytokines (IL-6 and IL-8) in PDLFs exposed to either *Escherichia coli* or *P. gingivalis* LPS (Supplementary Figures S1 and S2). Because the effect of *E. coli* LPS is known to differ from that of *P. gingivalis* LPS [37], we investigated cytokine production in PDLFs following stimulation by each LPS at various concentrations (1, 5, and 10 μg/mL) and different times. Furthermore, we found that 1 and 10 μg/mL, respectively, of crude *E. coli* and *P. gingivalis* LPS could effectively induce inflammatory cytokine expression in PDLFs. Notably, cytokines were produced by only crude *P. gingivalis* LPS stimulation (Toll-like receptor 2 (TLR2) and TLR4 agonists), but not by ultrapure *P. gingivalis* LPS (TLR4 agonist). This result is in accordance with the findings reported by Hirschfeld et al. [38]. We found that 100 µg/mL of LJLE significantly reduced the IL-6 and IL-8 expression induced by *E. coli* (1 µg/mL) or *P. gingivalis* LPS (10 µg/mL) at all subsequent time points (Figure 1). In addition, no cytotoxicity was observed following LJLE treatment of LPS-stimulated PDLFs (Supplementary Figure S3).

### 2.2. Inhibitory Effect of LJLE on Pro-Inflammatory Cytokine Expression in Mixed Red Complex Bacteria-Infected PDLFs

**Figure 2:**
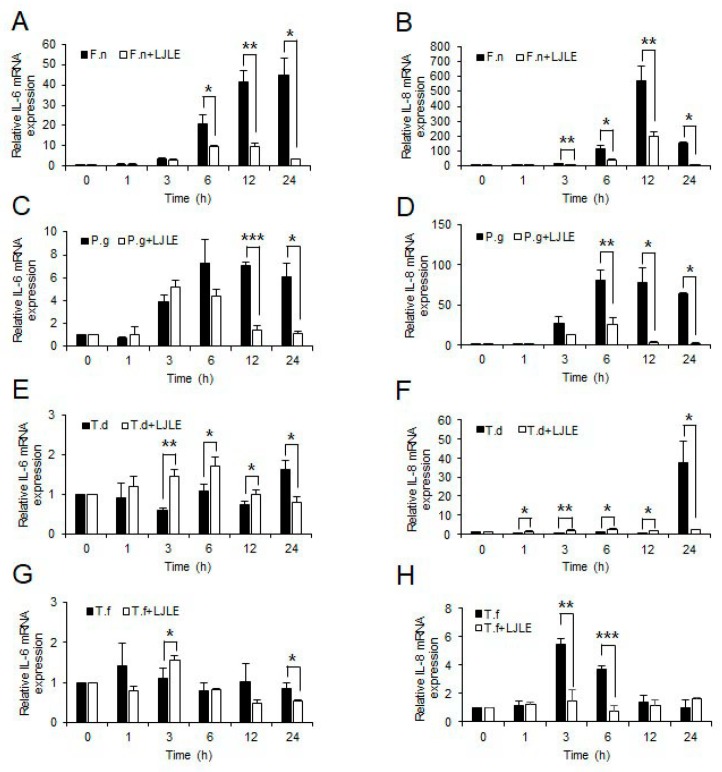
Inhibitory effect of LJLE on proinflammatory cytokine expression in human PDLFs infected with single species of oral bacteria at different time points (0, 1, 2, 3, 6, 12, and 24 h). IL-6 and IL-8 mRNA expression in human PDLFs infected with (**A**,**B**) *F. nucleatum* (F.n), (**C**,**D**) *P.* *gingivalis* (P.g), (**E**,**F**) *T. denticola* (T.d), and (**G**,**H**) *T. forsythia* (T.f) in the absence or presence of 100 μg/mL LJLE. Values are means ± standard deviation (SD) of triplicate assays. * *p* < 0.05, ** *p* < 0.01, and *** *p* < 0.001 (unpaired two-tailed Student’s *t*-test).

**Figure 3:**
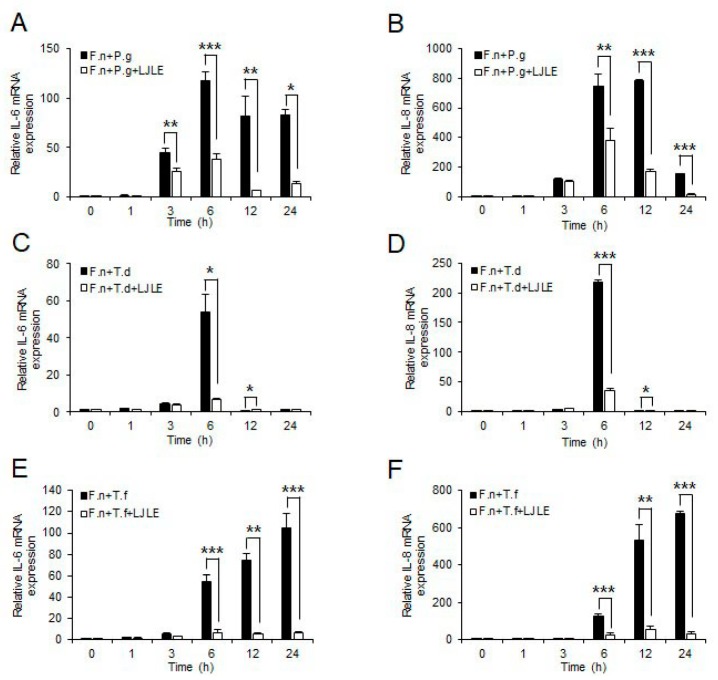
Inhibitory effect of LJLE on proinflammatory cytokine expression in human PDLFs infected with dual species of oral bacteria at different time points (0, 1, 2, 3, 6, 12, and 24 h). IL-6 and IL-8 mRNA expression in PDLFs infected with (**A**,**B**) *F. nucleatum* (F.n) plus *P. gingivalis* (P.g), (**C**,**D**) *F. nucleatum* plus *T. denticola* (T.d), and (**E**,**F**) *F. nucleatum* plus *T. forsythia* (T.f) in the absence or presence of 100 μg/mL LJLE. Values represent means ± standard deviation (SD) of triplicate assays. * *p* < 0.05, ** *p* < 0.01, and *** *p* < 0.001 (unpaired two-tailed Student’s *t*-test).

**Figure 4:**
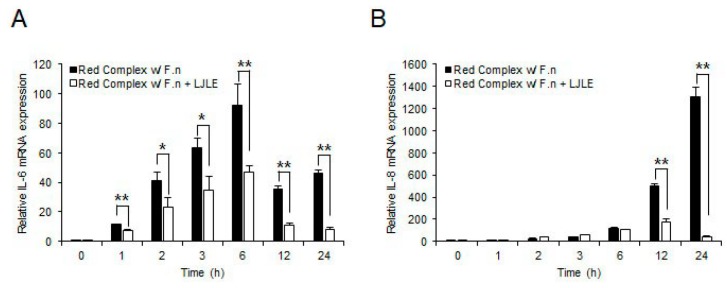
LJLE inhibits pro-inflammatory cytokine expression in mixed red complex bacteria, including *F. nucleatum* (F.n)-infected human PDLFs. Human PDLFs were stimulated with LPS in the absence or presence of 100 μg/mL LJLE for different times (0, 1, 2, 3, 6, 12, and 24 h). Real-time PCR was performed as described in the Materials and Methods. (**A**) Time-course of effects of LJLE on IL-6 and (**B**) IL-8 mRNA expression in mixed red complex (*P.* *gingivalis*, *T. denticola*, and *T. forsythia*), including *F. nucleatum* (F.n)-infected PDLFs. * *p* < 0.05 and ** *p* < 0.01 compared with bacterial infection alone (unpaired two-tailed Student’s *t*-test).

Next, to determine whether LJLE also suppresses pro-inflammatory cytokine expression by PDLFs after oral bacterial infection, we examined IL-6 and IL-8 expression levels in PDLFs treated with 100 µg/mL LJLE for varying times following infection with well-known periodontal pathogens (*P. gingivalis*, *T. denticola*, *T. forsythia*, and *F. nucleatum*). For bacterial infection, we attempted to use a combination of different strains. First, infection of PDLFs with a single bacterial strain revealed a relatively low cytokine production by the red complex strains (*P. gingivalis*, *T. denticola*, or *T. forsythia*) than that produced by *F. nucleatum*, and this effect was significantly decreased by LJLE treatment (Figure 2). Next, co-infection with each strain of the red complex bacteria with *F. nucleatum* led to a 10- to 100-fold increase in cytokine production, which was significantly decreased by LJLE treatment (Figure 3). Moreover, co-infection with all three strains of the red complex bacteria with *F. nucleatum* showed a remarkable increase in cytokine production (Figure 4), and treatment with LJLE significantly suppressed the IL-6 and IL-8 expression over the entire infection time course.

Because many studies have suggested that natural plant extracts show anti-inflammatory activity mediated by antioxidant or antimicrobial activity [39,40,41], we determined reactive oxygen species (ROS) generation and bacterial survival in *F. nucleatum*-infected PDLFs treated with LJLE. We found that ROS (H_2_O_2_) generation in *F. nucleatum*-infected PDLFs was not decreased by treatment with LJLE (Supplementary Figure S4), which also did not exhibit any bactericidal activity.

### 2.3. Dose-Dependent Inhibitory Effects of Pre- and Post-Treatment with LJLE on Pro-Inflammatory Cytokine Production in LPS- or F. nucleatum-Infected PDLFs

To determine whether LJLE suppresses inflammation in LPS- or *F. nucleatum*-infected PDLFs in a concentration-dependent manner, we evaluated the mRNA expression level of pro-inflammatory cytokines (IL-6 and IL-8) in LPS- or *F. nucleatum*-infected PDLFs following treatment with LJLE at different concentrations (0, 10, 50, and 100 μg/mL). Moreover, to ascertain whether the effect of LJLE was preventive or therapeutic, we treated PDLFs with LJLE at the indicated concentrations 2 h before or after infection with LPS or *F. nucleatum*. The incubation time for the treatment was determined based on the findings of previous studies on the effect of *L. japonica* fruit extract [42,43].

**Figure 5:**
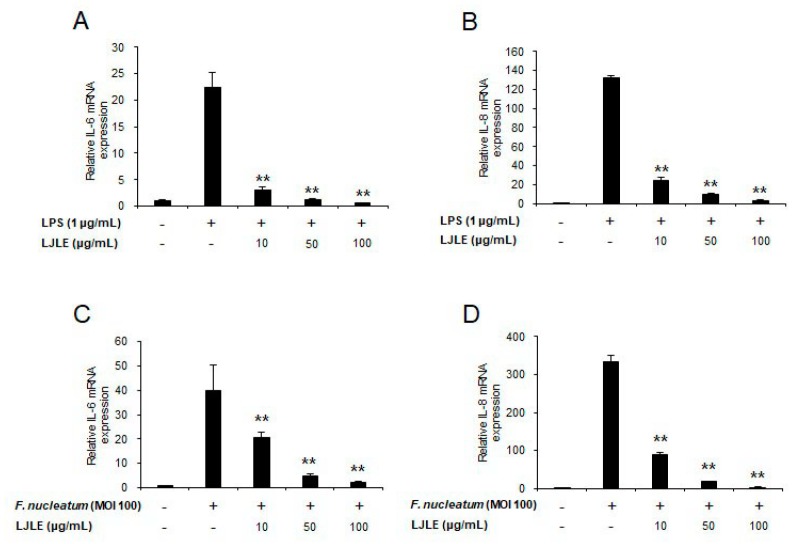
Pretreatment with LJLE inhibits pro-inflammatory cytokine expression in LPS or *F. nucleatum*-infected human PDLFs in a concentration-dependent manner. Human PDLFs were pretreated for 2 h with 0, 10, 50, or 100 μg/mL LJLE, followed by stimulation with *E. coli* LPS or *F. nucleatum* (MOI 100) for 24 h. Concentration-dependent inhibitory effect of LJLE pretreatment on IL-6 and IL-8 mRNA on (**A**,**B**) *E. coli* LPS-stimulated and (**C**,**D**) *F. nucleatum*-infected PDLFs. ** *p* < 0.01 compared with LPS or *F. nucleatum* alone (unpaired two-tailed Student’s *t*-tests).

First, pretreatment with LJLE significantly reduced the mRNA expression levels of IL-6 and IL-8 in a concentration-dependent manner (Figure 5). For example, the mRNA expression levels of IL-6 and IL-8 in LPS- and *F. nucleatum*-infected PDLFs were downregulated by approximately 5- and 3-fold, respectively, after treatment with 10 μg/mL LJLE. Moreover, the expression of these cytokines was completely blocked by pretreatment with 100 μg/mL LJLE.

Second, posttreatment with LJLE significantly reduced the mRNA expression levels of IL-6 and IL-8 in a concentration-dependent manner, but it was not as effective as pretreatment with LJLE. The mRNA expression levels of IL-6 and IL-8 in LPS-infected PDLFs were completely suppressed by 100 μg/mL LJLE (Supplementary Figure S5A,B). At the same concentration of LJLE in *F. nucleatum*-infected PDLFs, the expression of IL-6 and IL-8 was still approximately 21% and 7%, respectively, compared to the level of untreated cells (Supplementary Figure S5C,D).

**Figure 6:**
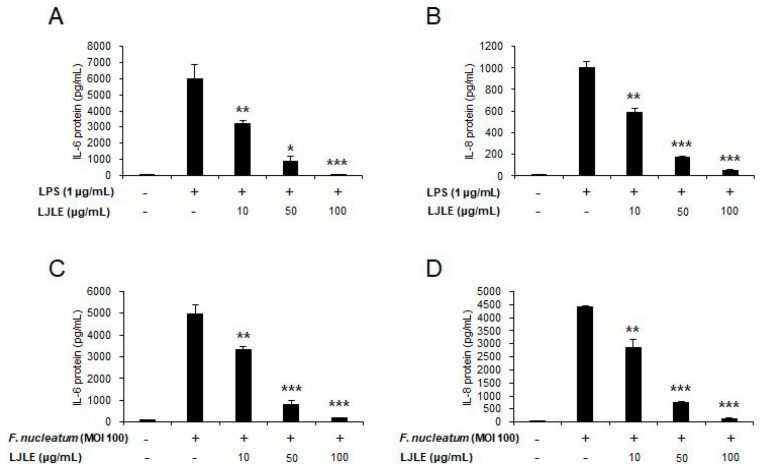
Pretreatment with LJLE inhibits pro-inflammatory cytokine secretion in LPS- or *F. nucleatum*-infected human PDLFs in a concentration-dependent manner. Human PDLFs were pretreated for 2 h with 0, 10, 50, or 100 μg/mL LJLE, followed by stimulation with *E. coli* LPS or *F. nucleatum* (MOI 100) for 24 h, and supernatants were analyzed using enzyme-linked immunosorbent assay (ELISA) to detect the presence of IL-6 and IL-8. Concentration-dependent inhibitory effect of LJLE pretreatment on IL-6 and IL-8 protein expression in (**A**,**B**) *E. coli* LPS-stimulated and (**C**,**D**) *F. nucleatum*-infected PDLFs. * *p* < 0.05, ** *p* < 0.01, and *** *p* < 0.001 compared with LPS or *F. nucleatum* alone (unpaired two-tailed Student’s *t*-test).

Next, to evaluate the effect of LJLE on protein expression levels of the pro-inflammatory cytokines, IL-6 and IL-8, in LPS- or *F. nucleatum*-infected PDLFs, we performed enzyme-linked immunosorbent assay (ELISA) to detect human IL-6 and IL-8 in cell culture supernatants. This experiment was also conducted using two different treatment methods, pre- and post-treatment, as described above. First, in the pretreatment setting, IL-6 and IL-8 protein production levels were decreased in LPS- or *F. nucleatum*-infected PDLFs in an LJLE concentration-dependent manner (Figure 6). Second, in the posttreatment setting, the protein levels of these cytokines were also significantly reduced by LJLE treatment, but this reduction was not as great as that following pretreatment (Supplementary Figure S6). Nevertheless, our results showed that, under both pretreatment and posttreatment conditions, LJLE effectively suppressed inflammatory cytokine production in LPS- or *F. nucleatum*-treated PDLFs.

### 2.4. Inhibitory Effect of LJLE on IL-1β-Induced Pro-Inflammatory Cytokine Production in PDLFs

**Figure 7:**
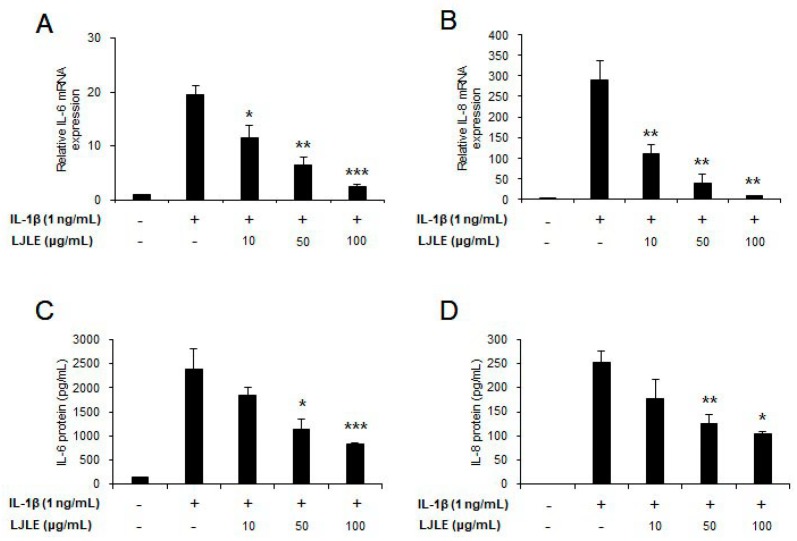
LJLE suppresses pro-inflammatory cytokine expression production in IL-1β-stimulated human PDLFs. Human PDLFs were treated for 12 h with 0, 10, 50, or 100 μg/mL LJLE in the absence or presence of 10 ng/mL IL-1β. Concentration-dependent enhancing effects of LJLE on IL-6 and IL-8 (**A**,**B**) mRNA and (**C**,**D**) protein in IL-1β-stimulated human PDLFs. * *p* < 0.05, ** *p* < 0.01, *** *p* < 0.001, and NS: Not significant compared with IL-1β alone (unpaired two-tailed Student’s *t*-test).

Inflammation is induced by stimulation with not only bacterial components (via TLR) but also IL-1β (via IL-1β signaling) in oral cells, and can exacerbate periodontal diseases [44]. Particularly, IL-1β is known to be a major mediator of periodontitis, and triggers the pro-inflammatory cytokines, IL-6 and IL-8, in gingival fibroblasts and PDLFs [21]. Thus, in this study, we assessed whether LJLE inhibits inflammation triggered by IL-1β stimulation in PDLFs. The expression of IL-6 and IL-8 mRNA was found to be significantly inhibited by LJLE in a concentration-dependent manner (Figure 7A,B). Accordingly, the protein expression levels of IL-6 and IL-8 were also significantly decreased by LJLE in a concentration-dependent manner (Figure 7C,D).

## 3. Discussion

The most common risk factor for periodontal disease is oral bacteria, which grow on biofilms in an interdependent manner on the tooth surface [45,46]. Several toxic substances, such as endotoxins, mucinous peptides, fatty acids, organic acids, hydrogen sulfide, and leukotoxin, were detected in the biofilm produced by oral bacteria [47]. Periodontal tissues secrete pro-inflammatory cytokines in response to these stimulants, which leads to the recruitment and activation of immune cells. However, excessive inflammation results in tissue damage and bone destruction [48]. Therefore, suppressing inflammation can be an effective strategy for the development of preventive and therapeutic approaches to reduce periodontal disease severity.

Periodontal diseases are characterized by the loss of PDL and alveolar bone in the periodontal tissue caused by bacterial infection. Therefore, limiting inflammation of bacteria-infected PDL is important to reduce the risk of periodontal disease. Numerous bacterial species are known to be associated with periodontal disease. In this study, we first investigated the effect of LJLE on proinflammatory cytokines in PDL after stimulation with *E. coli-* or *P. gingivalis*-LPS (Figure 1). We found that LJLE might inhibit TLR2/4-mediated cytokine production in PDL. Thus, we next determined the suppressive effect of LJLE on proinflammatory cytokines in PDL after infection of several oral bacterial strains. Previously, we reported that *F. nucleatum* likely interacted with gingival fibroblasts in the connective tissue, and provided a favorable environment for other anaerobic bacteria, such as *P. gingivalis* [14,15]. *F. nucleatum* is an orange complex bacterium, and its prevalence is significantly associated with increasing pocket depth [49]. It is a less pathogenic bacterium compared with the red complex bacteria, but it is a strong inducer of chemokines and proinflammatory mediators in oral cells [50]. In this study, we also observed higher cytokine production in the PDL by *F. nucleatum* than by any other bacterial strain (Figure 2A,B). Among four bacterial strains, *T. denticola* and *T. forsythia* induced a relatively lower level of proinflammatory cytokines than other strains (*F. nucleatum* and *P. gingivalis*) in the PDL (Figure 2E–H). Notably, all four bacterial strains induced a remarkable production of IL-8 than IL-6. In particular, IL-6 induction levels in the PDL by *T. denticola* or *T. forsythia* were considerably low (about 1−1.5 fold) and did not show a suppressive effect of LJLE, whereas the remarkably elevated IL-8 in the PDL by *T. denticola* or *T. forsythia* was significantly suppressed by LJLE treatment (Figure 2E–H).

Moreover, numerous studies have shown that red complex bacteria (*P. gingivalis*, *T. denticola*, and *T. forsythia*) show increased virulence characteristics compared with orange complex bacteria, such as *F. nucleatum*, during interaction with host cells [51]. We also observed considerable enhancement of proinflammatory cytokine production in PDL upon coinfection with the red complex bacteria, with *F. nucleatum* and LJLE treatment significantly suppressing IL-6 and IL-8 expression (Figure 3 and Figure 4).

Following stimulation with cytokines or bacteria, human primary oral cells secrete various inflammatory mediators, such as IL-6 and IL-8. IL-6 is an important mediator of the host response to injury and bacterial infection. It is known to be produced at considerably higher levels in patients with periodontitis than in healthy individuals. It is released from healthy human gingival fibroblasts following stimulation with oral pathogenic bacteria [14,15]. IL-8 is an important cytokine for maintaining a healthy periodontium because of its chemotactic activity that facilitates the infiltration of monocytes into periodontal tissues [49]. Moreover, IL-1β has been detected in the gingival fluid and tissues of patients with periodontal disease, but not in healthy individuals. It can also induce other inflammatory cytokines, such as IL-6 and IL-8, in gingival fibroblasts or PDL [52], which cause tissue destruction. Therefore, preventing excessive inflammation in periodontal tissues caused by various stimulants is important.

For the regulation of inflammation, antibiotics or non-steroidal anti-inflammatory drugs can be administered systemically. Non-steroidal anti-inflammatory drugs can cause various adverse effects in organs, including the gastrointestinal tract, nervous system, and liver [53]. The use of therapeutic systemic antibiotics has the potential to increase adverse effects and the incidence of several antibiotic-resistant strains [54]. Although oral bacteria are susceptible to various antibiotics, no single antibiotic that can inhibit all periodontal pathogens is available [55]. Oral cleansers can reduce gingivitis, but their efficacy in the prevention or treatment of periodontitis has not been established [56]. Moreover, local delivery of antimicrobial agents can enhance the suppression of subgingival pathogens [57]. Non-synthetic antibiotics, as well as phytochemicals, are used to treat periodontitis as adjuncts to nonsurgical treatment [58]. Therefore, exploring natural products, which are safer than conventional synthetic compounds, is recommended. Although the mechanisms by which natural products act are not well established, many studies have revealed their antioxidant activities [59] and other beneficial effects in the treatment of cancer [60,61,62], memory deficit, and Alzheimers [63]; atherosclerosis [64]; diabetes [65,66]; and other cardiovascular diseases [67]. Moreover, several plant-derived products have been suggested to have pharmacological effects in the prevention and treatment of periodontal diseases, such as the antibacterial effects of the extracts of maize [68], the anti-bone resorption activity of Magnoliae cortex extract [69], and the anti-inflammatory activity of elderberry [70].

In this study, we investigated the effect of LJLE on periodontal inflammation caused by bacterial infection or an inflammatory mediator. *L. japonica* has been used as a herbal medicine, and its fruit extract has been known to improve the symptoms of many diseases, including osteoarthritis [36], diabetes-induced retinal neurodegeneration [71], and blood-retinal barrier breakdown [72]. Recent studies about *L. japonica* have shown the effect of its fruit extract on inflammation in LPS-stimulated RAW264.7 cells [33,73,74,75,76]. One study suggested that the anti-inflammatory activity of *L. japonica* extract was due to its antioxidant activity, mediated by the inhibition of inducible nitric oxide synthase (iNOS) [74]. This study also showed the anti-inflammatory effect of LJLE on primary human oral cells triggered by various immunostimulants, including LPS, bacteria, and IL-1β. However, we did not observe any inhibitory effect of LJLE on iNOS expression and, in fact, there was no induction of iNOS expression in PDL after stimulation with bacteria or IL-1β. Moreover, we did not observe any reduction in ROS by LJLE treatment of PDLFs during bacterial infection. Thus, we believe that the anti-inflammatory effect of LJLE on PDLFs is not owing to its antioxidant activity and is not limited to only the TLR2/4-mediated inflammation, but also involves IL-1β-signaling-mediated inflammation. Finally, the anti-inflammatory effect of LJLE is both preventive and therapeutic in primary oral cells without cellular cytotoxicity. Taken together, these results indicate that LJLE might be a promising natural substance for improving periodontal health. Further studies are needed to confirm the in vivo efficacy of LJLE by using animal models of periodontal disease.

## 4. Materials and Methods

### 4.1. Ethics Statement

The isolation of human PDLFs was approved by the Chonnam National University Dental Hospital Institutional Review Board (Approval No., CNUDH-2016-013; 20 October 2016). Written informed consent was obtained from all subjects after the nature and possible consequences of the studies were explained to them. All participants were adults who did not have periodontal disease.

### 4.2. Preparation of LJLE

The leaves and stems of *L. japonica* used in this experiment were collected from Jeju Island in Korea by the Jeju Biodiversity Research Institute, Jeju Technopark. The dried *L. japonica* leaves (100 g) were extracted with 80% ethanol three times for 24 h each at room temperature [76]. The lyophilized extract was sonicated six times for 8 s in 1 mL of 80% ethanol, mixed thoroughly, and stored in a refrigerator at −20 °C until use.

### 4.3. Bacterial Strains and Culture Condition

The *F. nucleatum* subspecies *polymorphum* American Type Culture Collection (ATCC) 10953^T^, *P. gingivalis* KCOM 2804, *T. denticola* ATCC 35405^T^, and *T. forsythia* ATCC 43037^T^ were provided by the Korean Collection for Oral Microbiology (Gwangju, Korea) and purchased from ATCC (Manassas, VA, USA). Briefly, bacterial cultures were prepared by culturing *P. gingivalis* and *F. nucleatum* in medium containing 0.5% yeast extract, 0.05% cysteine HCl-H_2_O, 10 μg/mL hemin, and 2 μg/mL vitamin K1 in Tryptic Soy Broth. *T. forsythia* was cultured by adding 10 μg/mL *N*-acetylmuraminic acid (Sigma, St. Louis, MO, USA) to the above medium. *T. denticola* was added to the heart infusion broth with 1.0% trypticase peptone, 0.25% yeast extract, 0.05% sodium thioglycollate, 0.1% l-cysteine HCl-H_2_O, 0.025% l-asparagine, 0.2% d-glucose, 0.0001% resazurin, 0.20025% sodium bicarbonate, 2% rabbit serum, and 2% thiamine pyrophosphate (TPP)/volatile fatty acid solution (0.03% TPP, 0.05% isobutyric acid, 0.05% 2-methylbutyric acid, 0.05% isovaleric acid, 0.05% valeric acid, and 0.098 N sodium hydroxide). These strains were cultured under anaerobic conditions (10% H_2_, 5% CO_2_, and 85% N_2_) in a 37 °C anaerobic chamber (Bactron I; Sheldon Manufacturing Inc., Cornelius, OR, USA) [14]. The culture broth absorbance was measured at 600 nm, and the number of bacteria was counted. The bacteria were collected by centrifugation (7000× *g*, 10 min), and washed in phosphate-buffered saline before the cell suspension density was adjusted to 2 × 10^7^ colony forming unit (CFU)/5 μL in the buffer as the multiplicity of infection (MOI) of 200. Cells were incubated for the appropriate time and then washed twice with PBS.

### 4.4. Culture of Primary PDLFs

Primary human PDLFs were prepared as described by Somerman et al. [77]. Collection of human PDL from the premolar and third molar was approved by the Institutional Review Board of Chonnam National University Dental Hospital (Approval No., CNUDH-2016-013; 20 October 2016). All participants were adults without periodontal disease. The PDLFs were grown in Dulbecco’s modified Eagle’s medium (Gibco BRL, Waltham, MA, USA) supplemented with 10% heat-inactivated fetal bovine serum (GenDepot, Katy, TX, USA), 100 U/mL penicillin, and 100 μg/mL streptomycin (GenDepot, USA) at 37 °C in a humidified atmosphere containing 5% CO_2_. When they achieved confluence, the cells were trypsinized using 0.25% trypsin/0.02% ethylenediaminetetraacetic acid solution (Sigma-Aldrich, St. Louis, MO, USA). Cells in which PDLFs proliferated sufficiently were sub-cultured five to six times at a ratio of 1:2 to 1:3 in a 100 mm culture dish and were used in this experiment.

### 4.5. Measurement of Cell Viability

To evaluate the cytotoxicity of LJLE, we used the 3-(4,5-dimethylthiazol-2-yl)-2,5-diphenyltetrazolium bromide (MTT) colorimetric assay to measure cell viability after treatment with inhibitors [78]. PDLFs were seeded into 96-well plates at a density of 10^4^ cells/well in DMEM containing 10% fetal bovine serum. The cells were incubated with an appropriate concentration of the extract with *E. coli* or *P. gingivalis* LPS at the indicated concentration and time, after which the medium was carefully replaced with fresh medium containing MTT (EZ-Cytox; DoGenBio, Gwangju, Korea) solution, and the incubation was continued for an additional 3 h at 37 °C in an atmosphere of 5% carbon dioxide. The same volume of 10% sodium dodecyl sulfate in 0.01 M hydrochloric acid solution was added to each well, followed by thorough mixing using a pipette, and then the incubation was continued for another 3 h. The absorbance at 450 nm was measured using a microplate reader (Bio-Rad Laboratories, Hercules, CA, USA). Reported values are the means of triplicates (one line of 96-well microplate) and are expressed as a percentage of control values (A450 of inhibitor/A450 of control) × 100.

### 4.6. Quantitative Real-Time PCR

Total RNA was isolated using RNeasy kits (Qiagen, Hilden, Germany) primed with random hexamer oligonucleotides and was reverse transcribed using a PrimeScript RT Reagent kit (Takara Biotechnology, Tokyo, Japan). Real-time quantitative PCR was performed using SYBR Green Master Mix (Takara Biotechnology, Tokyo, Japan) and the following primers: Human IL-6 F: 5′-agggctcttcggcaaatgta-3′ and R: 5′-tgcccagtggacaggtttc-3′, and human IL-8 F: 5′-tttctgttaaatctggcaaccctagt-3′ and R: 5′-ataaaggagaaaccaaggcacagt-3′. All data were normalized to human glyceraldehyde 3-phosphate dehydrogenase (GAPDH).

### 4.7. ELISA

IL-6 and IL-8 concentrations in the secreted cell culture supernatants were quantified using commercially available human-specific ELISA kits (Biolegend, San Diego, CA, USA). Briefly, 96-well plates were coated with anti-human IL-6 or IL-8 monoclonal antibodies. After blocking with the assay solution for 2 h to avoid non-specific binding, 100 μL of the standard IL-6, IL-8, or culture supernatants were added. The cytokines were detected using a horseradish peroxidase-labeled monoclonal antibody to each target protein after 100 μL of the anti-human biotinylated antibodies were added to each well and incubated for 2 h at 21 °C. The microplate was washed to remove the unbound enzyme-labeled antibodies. The amount of horseradish peroxidase bound to each well was determined by adding the substrate solution. The reaction was stopped by the addition of sulfuric acid, and the plates were read at 450 nm by using a SpectaMax i3X micro-titer plate reader (San Jose, CA, USA). The concentrations of each target were determined by interpolation from a standard curve and presented as picograms per milliliters (pg/mL ± one standard error of the mean).

### 4.8. Statistical Analysis

Statistical analysis was performed using one-way analysis of variance (ANOVA) with Tukey’s test by using the Statistical Packages for Social Science (SPSS, 23.0, Chicago, IL, USA). Furthermore, *p*-values < 0.05 were considered statistically significant and are indicated by asterisks in the figures. Values are the means ± SD (indicated by error bars) of three independent experiments performed in triplicate.

## 5. Conclusions

In this study, we found that LJLE is effective for the suppression of mRNA and protein expression of proinflammatory cytokines (IL-6 and IL-8) induced by various inflammatory stimuli, including LPS, oral pathogens, and IL-1β, in the PDL after both pre- and post-treatments. The results of this study suggest that LJLE could possibly be used for the prevention and treatment of inflammatory periodontal disease.

## Supplementary Materials

Supplementary materials can be found at http://www.mdpi.com/1422-0067/19/9/2494/s1.

## Abbreviations

PDL	Periodontal ligament
PDLFs	Periodontal ligament fibroblasts
LJLE	*Litsea japonica* leave extract
LPS	Lipopolysaccharide
IL-6	Interleukin-6
IL-8	Interleukin-8
SD	Standard deviation
TLR	Toll-like receptor
mRNA	Messenger ribonucleic acid
IL-1β	Interleukin-1 beta
ROS	Reactive oxygen species
iNOS	Inducible nitric oxide synthase
MOI	Multiplicity of infection
TPP	Thiamine pyrophosphate
CFU	Colony forming unit
MTT	3-(4,5-Dimethylthiazol-2-yl)-2,5-diphenyltetrazolium bromide
PCR	Polymerase chain reaction
GAPDH	Glyceraldehyde 3-phosphate dehydrogenase
ELISA	Enzyme-linked immunosorbent assay
DCF	Dichlorofluorescein fluorescence
