# Supplementary material for: Litsea japonica Leaf Extract Suppresses Proinflammatory Cytokine Production in Periodontal Ligament Fibroblasts Stimulated with Oral Pathogenic Bacteria or Interleukin-1β
Source: Int J Mol Sci. 2018 Aug 23;19(9):2494. doi: 10.3390/ijms19092494 (PMC6163774; doi:10.3390/ijms19092494)
Supplement: Supplementary file 1 [file ijms-19-02494-s001.pdf]

Supplemental Figure S1

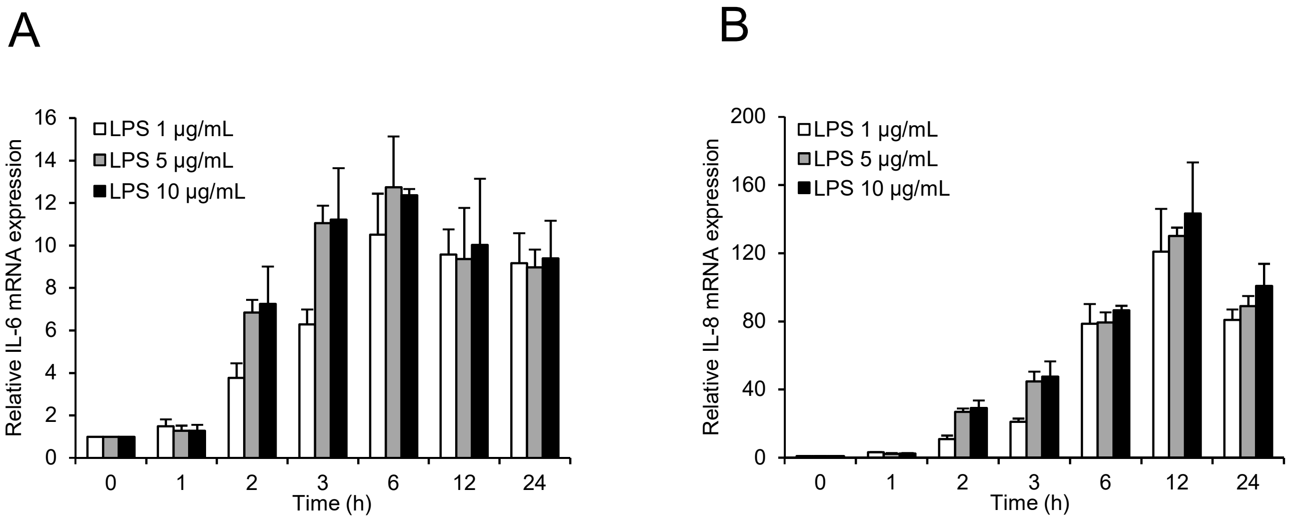

Supplementary Figure 1. Proinflammatory cytokine expression in human PDLFs stimulated with Esche richia coli lipopolysaccharide (LPS; 1, 5, and 10 μg/mL) for varying times (0, 1, 2, 3, 6, 12, and 24 h). (A) I L-6 and (B) IL-8 mRNA expression in E. coli LPS-stimulated human PDLFs.

Supplemental Figure S2

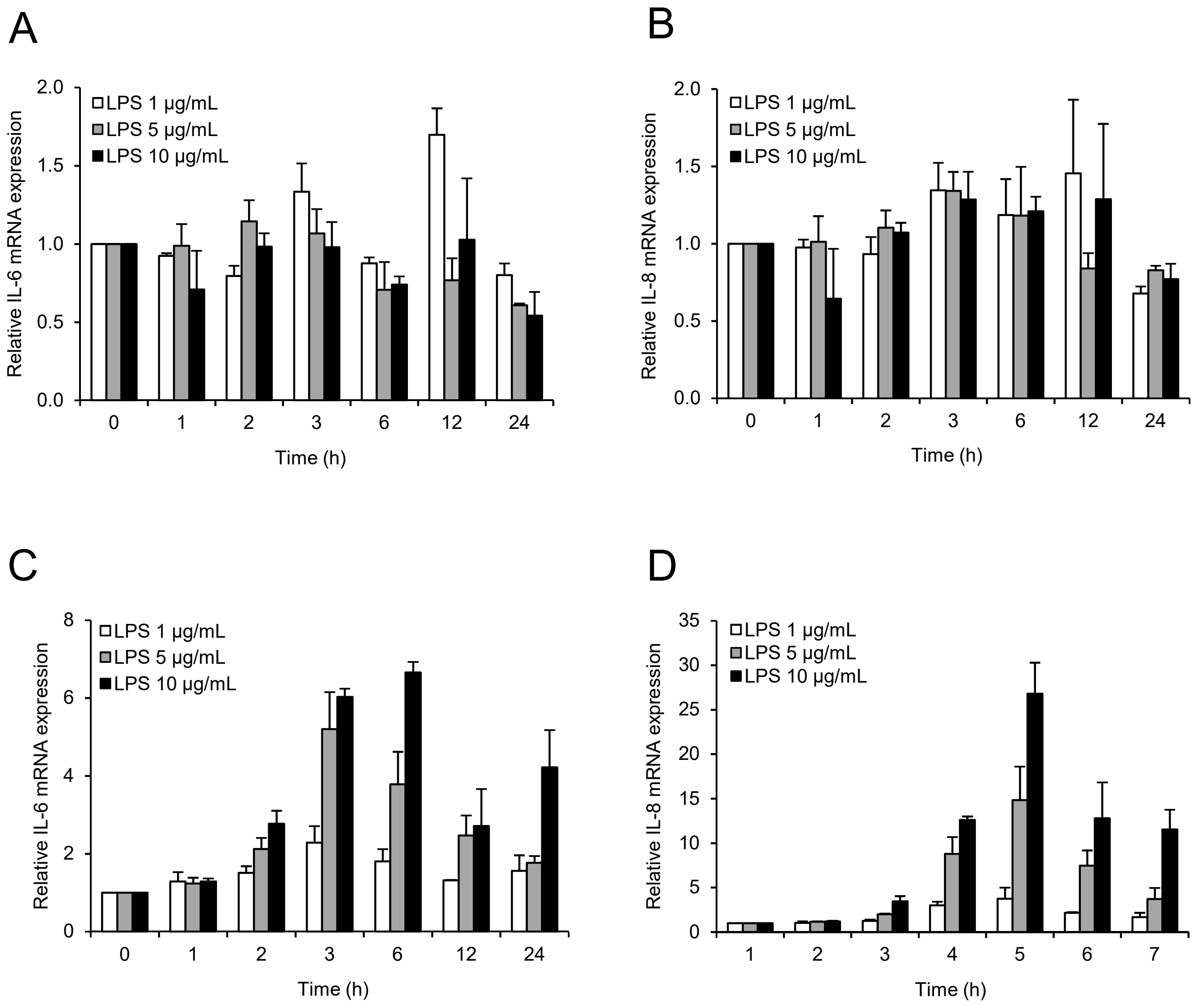

Supplementary Figure 2. Proinflammatory cytokine expression in human PDLFs stimulated with *Porphyromo nas gingivalis* lipopolysaccharide (LPS) with two grades of purity (1, 5, and 10 μg/mL) for varying times (0, 1, 2, 3, 6, 12, and 24 h). IL-6 and IL-8 mRNA expression in (A and B) ultrapure and (C and D) standard *P. gingiva lis* LPS-stimulated human PDLFs. Values are means ± SD of triplicate assays. Student's *t*-test was used to com pare values between groups.

Supplemental Figure S3

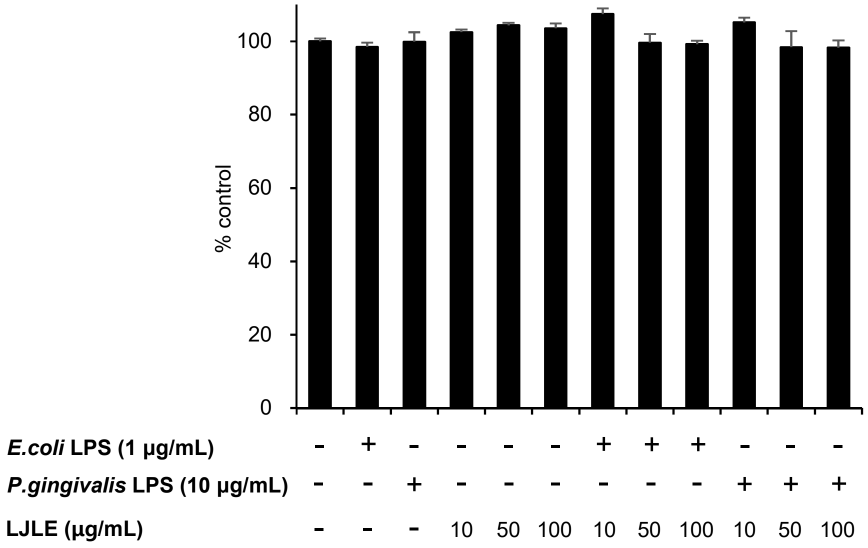

Supplementary Figure 3. Effects of LJLE on the viability of unstimulated and LPS-stimulated PDLFs. PDLFs were incubated for 12 h without (control) or with LPS in the presence of the indicated concent rations of LJLE, and the relative viable cell number was determined using the MTT method. Values a re means ± SD of triplicate assays.

Supplemental Figure S4

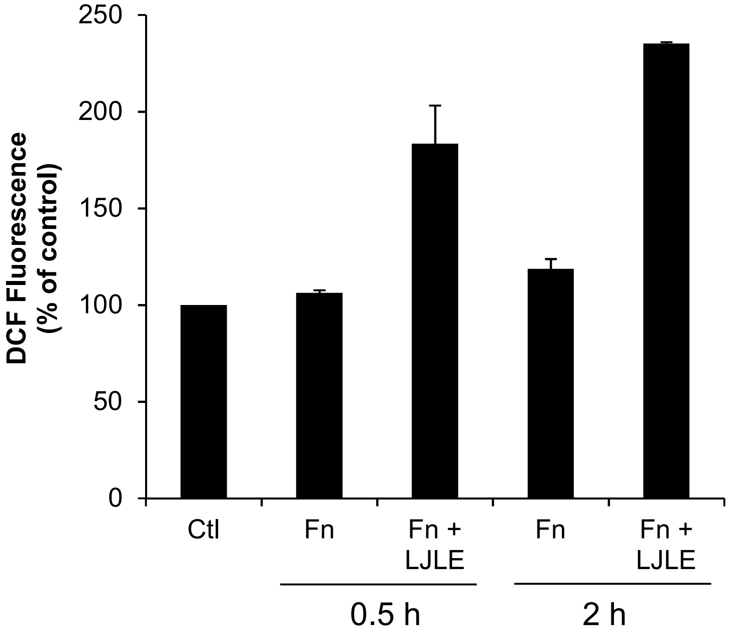

Supplementary Figure 4. Reactive oxygen species (ROS) production in *F. nucleatum*-infected human PD LFs in the absence or presence of 100 μg/mL LJLE at different time points. Hydrogen peroxide level wa s measured in PDLFs by using dichlorofluorescein fluorescence (DCF) after 0.5 h and 2 h of infection w ith *F. nucleatum*. Levels of DCF fluorescence were compared with that of uninfected control (100%). Val ues are means ± SD of triplicate assays.

Supplemental Figure S5

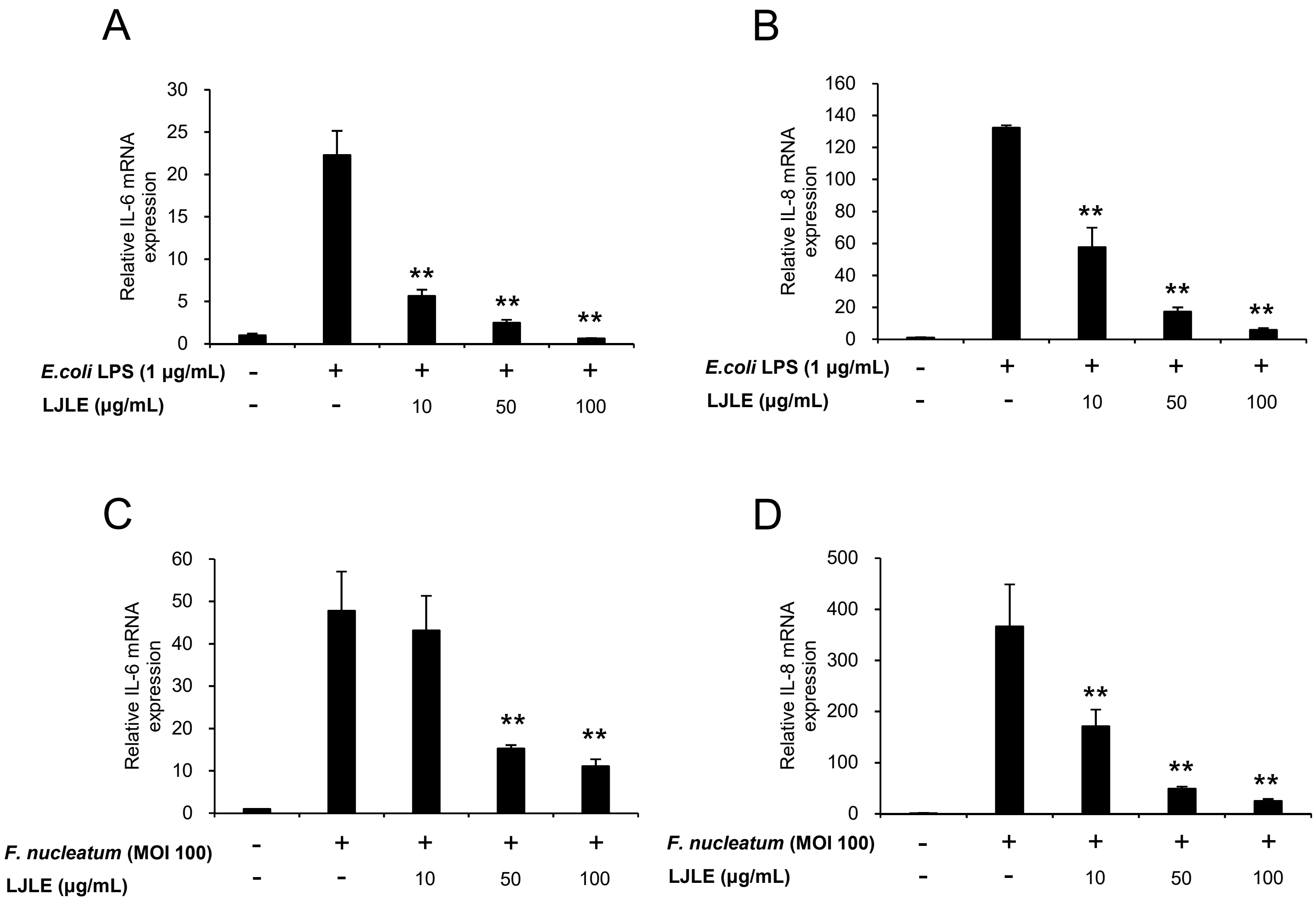

Supplementary Figure 5. Posttreatment of LJLE inhibits pro-inflammatory cytokine expression in LPS or *F. nucleatu m*-infected human PDLFs in a concentration-dependent manner. Human PDLFs were stimulated with *Escherichia c oli* LPS or *F. nucleatum* for 2 h and then post-treated with 0, 10, 50, or 100 μg/mL LJLE for 24 h. Concentration-depe ndent inhibitory effect of LJLE posttreatment on IL-6 and IL-8 mRNA in (A and B) *E. coli* LPS-stimulated and (C an d D) *F. nucleatum*-infected PDLFs. *$P < 0.05$ and **$P < 0.01$ compared with LPS or *F. nucleatum* alone (unpaired two-t ailed Student's *t*-test).

Supplemental Figure S6

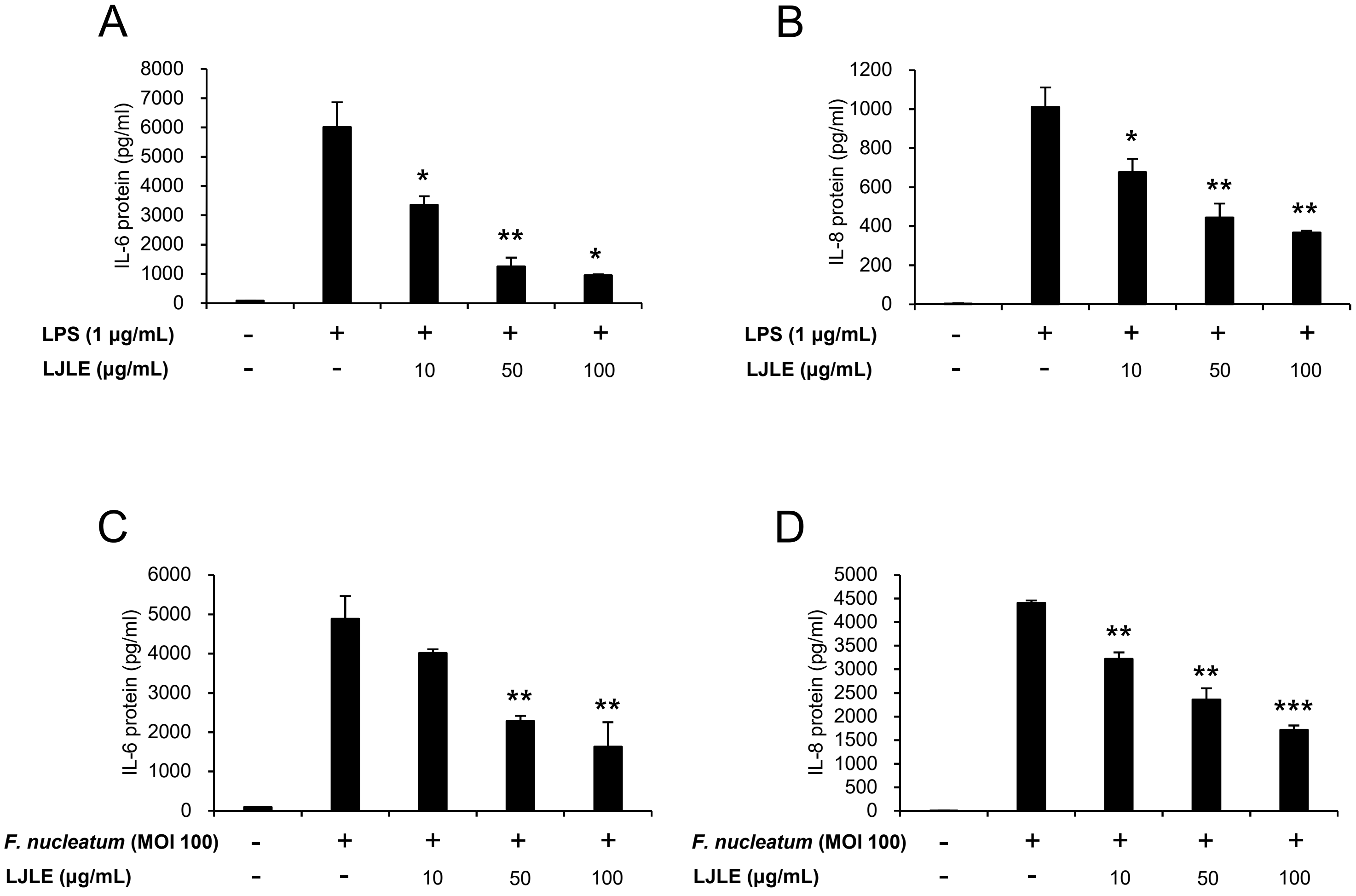

Supplementary Figure 6. Posttreatment with LJLE inhibits pro-inflammatory cytokine secretion in LPS or *F. nucleatum*-infected human PDLFs in a concentration-dependent manner. Human PDLFs were stimulated with *E. coli* LPS or *F. nucleatum* for 2 h and then post-treated with 0, 10, 50, or 100 μg/mL LJLE for 24 h, and supernatants were analyzed using ELISA to detect interleukin (IL)-6 and IL-8 expression. Concentration-dependent inhibitory effect of LJLE posttreatment on IL-6 and IL-8 protein in (A and B) *E. coli* LPS-stimulated PDLFs and (C and D) *F. nucleatum*-infected PDLFs. *$P < 0.05$ and **$P < 0.01$ compared with LPS or *F. nucleatum* alone (unpaired two-tailed Student's *t*-test).
